# Determinants and management approaches for fear of disease progression among stroke survivors

**DOI:** 10.1097/MD.0000000000048194

**Published:** 2026-04-03

**Authors:** Xu Liu, Lianhua Qin, Jieping Zhong

**Affiliations:** a Department of Neurology, Chengdu Shang Jin Nan Fu Hospital, Chengdu, Sichuan Province, China; b West China Hospital, Sichuan University, Chengdu, Sichuan Province, China.

**Keywords:** fear of disease progression, Fear of Progression Questionnaire-Short Form, management approaches, risk factors, stroke

## Abstract

This study aimed to identify risk factors for fear of disease progression (FoP) among stroke survivors and propose management strategies. A cross-sectional study of 149 stroke patients (February 2022–March 2025) was conducted using the Fear of Progression Questionnaire-Short Form, Pittsburgh Sleep Quality Index, Social Support Rating Scale, and Family Adaptability and Cohesion Evaluation Scale II-Chinese Version. Pearson correlations and logistic regression were applied. Clinically significant FoP (≥34) was observed in 45.64% of patients. FoP positively correlated with poor sleep quality and negatively correlated with social support and family functioning (*P* < .001). Multivariate analysis identified neurological deficit severity, comorbidities, unemployment, and sleep disturbance as risk factors, while older age and higher social support were protective factors. FoP is prevalent among stroke survivors and is associated with sleep quality and social support. Targeted psychosocial and sleep-focused interventions may help reduce FoP.

## 1. Introduction

With global costs exceeding $721 billion, stroke is a primary contributor to global mortality (ranked second) and disability (ranked third).^[1]^ Ischemic strokes (≈65%) are the predominant form, compared to hemorrhagic strokes (≈35%, including intracerebral and subarachnoid hemorrhage). Epidemiologically, ischemic strokes are more prevalent in high-income countries (74.9% vs 63.4%), while middle- and low-income nations exhibit a relatively higher share of hemorrhagic strokes (31.1% vs 17.8%).^[2]^ Stroke is initiated by a disruption in cerebral blood flow and oxygenation, which induces cortical injury. This injury, in turn, gives rise to varying degrees of impairment in motor, cognitive, swallowing, and speech functions.^[3,4]^ Such impairments frequently curtail activities of daily living and are also associated with long-term negative consequences for mental health.^[5]^ Conceptualized as a stressor, fear of disease progression (FoP) refers to the biopsychosocial repercussions triggered by concerns about illness worsening or relapse.^[6]^ At moderate intensities, it can serve as a catalyst for positive health actions. When reaching excessive levels, however, it produces counterproductive effects, often resulting in psychological distress, a decline in quality of life, hazardous drinking patterns, and failure to follow prescribed treatments.^[7]^ Fear of disease progression has been shown to substantially affect the symptom burden experienced by recovering stroke patients, highlighting the potential benefit of interventions targeting this fear.^[8]^ Systematic reviews report a 45.0% to 66.0% FoP risk in Chinese stroke patients, possibly influenced independently by social support and family dynamics.^[9]^ Beyond stroke, Tian and Wang^[10]^ established a clear link between FoP and impaired sleep quality among hematologic cancer patients. This study is based on the hypothesis that FoP in stroke survivors is significantly correlated with sleep disorders, social support, and family cohesion and adaptability. A further objective is to delineate risk factors that predispose patients to such fears, thereby supporting the design of evidence-based management approaches. Given the scarcity of existing research in this area, the findings are expected to inform and refine clinical practices for stroke survivors.

## 2. Materials and methods

### 2.1. Case selection

This study was approved by the Ethics Committee of Chengdu Shang Jin Nan Fu Hospital. Inclusion required a clinical and radiographic stroke diagnosis,^[11]^ age ≥ 18 years, presentation ≤ 14 days post-onset, absence of cognitive impairment or severe aphasia, and availability of complete medical records. Exclusions encompassed severe disability or hemiplegia, pregnancy/lactation, a modified Rankin Scale score ≥ 4,^[12]^ diagnosis of malignant tumor, or severe organ dysfunction.

With the approval of the institutional ethics committee, this study recruited 149 stroke patients. These participants were selected from admissions between February 2022 and March 2025 after rigorous assessment against the aforementioned criteria. The required sample size was estimated using G*Power 3.1 software (Heinrich-Heine-Universität Düsseldorf, Düsseldorf, Germany). Based on previous studies reporting medium effect sizes (odds ratio [OR] ≈ 2.0–3.0) for psychosocial predictors of FoP in chronic disease populations, assuming α = 0.05, power (1 − β) = 0.80, and 6 predictors in the logistic regression model, the minimum required sample size was calculated to be 134 participants. Considering a potential 10% attrition or incomplete response rate, a target sample size of at least 148 was determined. The final sample of 149 patients therefore met the statistical power requirements.

### 2.2. Investigation methods

Stroke patients were evaluated within 48 to 72 hours of admission, once their medical status was stable. In a tranquil, private setting, a graduate nursing student and a stroke rehabilitation specialist nurse administered the Fear of Progression Questionnaire-Short Form (FoP-Q-SF), Pittsburgh Sleep Quality Index (PSQI), Social Support Rating Scale (SSRS), and Family Adaptability and Cohesion Evaluation Scale II-Chinese Version (FACESII-CV). All assessors participated in unified training before study commencement. Patients gave written consent after being fully informed about the research objectives and methodology. They were also guided on how to complete the questionnaires, with the principles of anonymity and non-maleficence emphasized. Patients unable to self-report were assisted by a stroke rehabilitation nurse who transcribed their verbal answers. All questionnaires underwent immediate collection and verification. Validity was contingent upon fully completed items and consistent responses. The questionnaire captured patient identifiers (name and hospitalization number) and scale responses, with all fields being mandatory. Submission was restricted to 1 entry per device, mobile number, or IP address, with a 25-minute time constraint.

### 2.3. Data collection and outcome measurement

#### 2.3.1. FoP

Fear of disease progression quantification employed the FoP-Q-SF,^[13]^ a brief 12-item measure using 5-point response options (total score range: 12–60). Ascending scores reflect greater fear intensity, with scores ≥ 34 reflecting clinically significant fear. The measure’s reliability in our sample was confirmed by a Cronbach α value of 0.76.

#### 2.3.2. Sleep quality

An assessment of sleep quality was conducted utilizing the PSQI.^[14]^ The 18-item questionnaire is structured across 7 domains: sleep quality, sleep latency, sleep duration, sleep efficiency, sleep disturbances, use of sleep medications, and daytime dysfunction. A global score (range: 0–21) is derived from the sum of item scores (0–3 per item), with increased scores signifying inferior sleep quality. The reliability analysis for the PSQI in this sample showed a Cronbach α of 0.842.

#### 2.3.3. Social support

Using the SSRS,^[15]^ social support was quantified across 3 facets: objective, subjective, and utilization of support. Elevated scores correspond to stronger social support. Reliability analysis indicated a Cronbach α of 0.891.

#### 2.3.4. Family cohesion and adaptability

The evaluation of family cohesion and adaptability was conducted with the FACESII-CV.^[16]^ The 30-item scale contains a 16-item Cohesion subscale (score range: 25–77) and a 14-item Adaptability subscale (score range: 13–65). Respondents indicate their level of agreement on a 5-point scale (1 = least to 5 = most), where elevated scores reflect greater levels of each construct. The FACESII-CV has established reliability and validity for Chinese populations, evidenced by a Cronbach α coefficient of 0.901.

### 2.4. Statistical methods

Continuous variables are summarized as means with standard deviations ($\bar{x}\pm s$), compared between groups by independent *t* tests and within groups by paired *t* tests. For categorical variables, frequencies (percentages) are provided, and group differences were evaluated with the chi-square test. Statistical processing was carried out with SPSS (Statistical Package for the Social Sciences) 21.0 (IBM, SPSS, Chicago). This study employed Pearson *r* to analyze associations of FoP-Q-SF with PSQI, SSRS, and FACESII-CV. To identify risk factors for FoP, variables were first screened by univariate analysis; significant variables were subsequently included in a binary logistic regression model. *P*-values below .05 were deemed statistically significant.

## 3. Results

### 3.1. FoP-Q-SF scores in 149 stroke cases

The FoP-Q-SF measured FoP in 149 stroke patients, yielding a mean score of 32.32 ± 7.90. Of these, 68 patients (45.64%) scored ≥34, reflecting clinically significant fear. Data are summarized in Table 1.

**Table 1:** FoP-Q-SF results for the stroke cohort.

Indicators	n = 149
FoP-Q-SF (points)	32.32 ± 7.90
≥34	68 (45.64)
<34	81 (54.36)

FoP-Q-SF = Fear of Progression Questionnaire-Short Form.

### 3.2. PSQI of 149 stroke patients

Evaluation of sleep quality among 149 stroke patients, conducted with the PSQI, yielded a mean global score of 9.67 ± 2.00 points. Table 2 summarizes the scores corresponding to each PSQI dimension.

**Table 2:** PSQI assessment in the study cohort.

Indicators	n = 149
Sleep quality (points)	1.48 ± 0.62
Sleep latency (points)	1.13 ± 0.64
Sleep duration (points)	1.28 ± 0.65
Sleep efficiency (points)	1.95 ± 0.86
Sleep disturbances (points)	1.65 ± 0.77
Use of sleep medications (points)	0.53 ± 0.50
Daytime dysfunction (points)	1.67 ± 0.82
Global PSQI (points)	9.67 ± 2.00

PSQI = Pittsburgh Sleep Quality Index.

### 3.3. SSRS assessment in 149 stroke cases

Based on SSRS assessments for 149 stroke patients, the mean total social support score was (31.31 ± 4.11) points. Dimension-specific scores are detailed in Table 3. Notably, nearly all participants (97.99%) fell into the moderate social support category.

**Table 3:** Distribution of SSRS scores in 149 stroke patients.

Indicators	n = 149
Objective support (points)	9.32 ± 2.09
Subjective support (points)	16.93 ± 3.04
Utilization of support (points)	5.06 ± 1.16
SSRS (points)	31.31 ± 4.11
Low support	3 (2.01)
Moderate support	146 (97.99)
High support	0 (0.00)

SSRS = Social Support Rating Scale.

### 3.4. FACESII-CV evaluation in 149 stroke patients

Based on FACES II-CV assessments, 149 stroke patients reported mean family cohesion and adaptability scores of 43.22 ± 6.54 and 38.34 ± 6.43 points, respectively (see Table 4 for specifics).

**Table 4:** FACES II-CV measurements of 149 stroke patients.

Indicators	n = 149
Family cohesion (points)	43.22 ± 6.54
Family adaptability (points)	38.34 ± 6.43

FACESII-CV = Family Adaptability and Cohesion Evaluation Scale II-Chinese Version.

### 3.5. Bivariate correlations: FoP-Q-SF with PSQI, SSRS, and FACESII-CV

**Figure 1.**
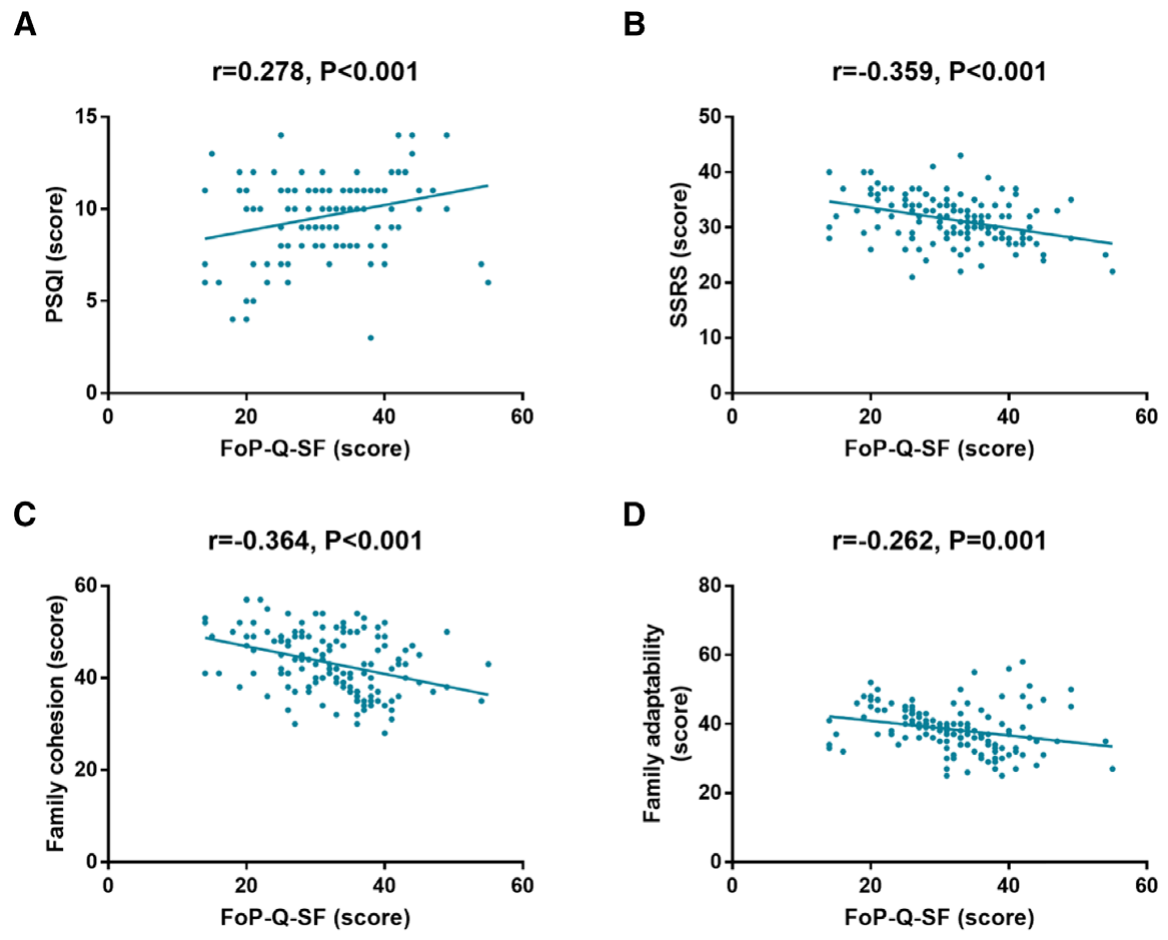
Correlation of FoP-Q-SF with PSQI, SSRS, and FACESII-CV. (A) FoP-Q-SF and PSQI association. (B) FoP-Q-SF and SSRS correlation. (C) FoP-Q-SF correlation with family cohesion. (D) FoP-Q-SF correlation with family adaptability. FACESII-CV = Family Adaptability and Cohesion Evaluation Scale II-Chinese Version, FoP-Q-SF = Fear of Progression Questionnaire-Short Form, PSQI = Pittsburgh Sleep Quality Index, SSRS = Social Support Rating Scale.

Using Pearson *r*, we evaluated how the FoP-Q-SF related to the PSQI, SSRS, and FACESII-CV. The FoP-Q-SF score rose with the increasing PSQI score (*r* = 0.278, *P* < .001). In contrast, significant negative correlations were observed with the SSRS score (*r* = −0.359, *P* < .001), as well as with the FACESII-CV family cohesion (*r* = −0.364, *P* < .001) and family adaptability (*r* = −0.262, *P* < .001) subscales. These relationships are further detailed in Figure 1.

### 3.6. Determinants of FoP: univariate screening

The FoP group consisted of 68 individuals with FoP-Q-SF scores ≥34, and the remaining formed the non-FoP group. According to the univariate analysis summarized in Table 5, significant factors included age, neurological deficit severity, comorbidities, occupational status, PSQI, SSRS, family cohesion, and family adaptability (*P* < .05). Gender, stroke type, initial episode status, and education level were not significantly correlated (*P* > .05).

**Table 5:** Predictors of FoP among stroke patients: univariate findings.

Indicators	FoP (n = 68)	Non-FoP (n = 81)	χ^2^/*t*	*P*
Age (yr)			6.526	.011
<60 (n = 60)	35 (51.47)	25 (30.86)		
≥60 (n = 89)	33 (48.53)	56 (69.14)		
Sex			1.528	.216
Male (n = 92)	32 (47.06)	30 (37.04)		
Female (n = 87)	36 (52.94)	51 (62.96)		
Stroke type			0.826	.364
Ischemic (n = 127)	56 (82.35)	71 (87.65)		
Hemorrhagic (n = 22)	12 (17.65)	10 (12.35)		
Neurological deficit severity			9.318	.002
Mild (n = 108)	41 (60.29)	67 (82.72)		
Moderate (n = 41)	27 (39.71)	14 (17.28)		
Comorbidities			11.384	<.001
<2 (n = 100)	36 (52.94)	64 (79.01)		
≥2 (n = 49)	32 (47.06)	17 (20.99)		
Initial episode			1.997	.158
Yes (n = 105)	44 (64.71)	61 (75.31)		
No (n = 44)	24 (35.29)	20 (24.69)		
Education level			1.955	.162
≥Senior high school (n = 85)	43 (63.24)	42 (51.85)		
<Senior high school (n = 64)	25 (36.76)	39 (48.15)		
Occupational status			6.276	.012
In-service (n = 67)	23 (33.82)	44 (54.32)		
Unemployed (n = 82)	45 (66.18)	37 (45.68)		
PSQI (points)			9.387	.002
<9 (n = 40)	10 (14.71)	30 (37.04)		
≥9 (n = 109)	58 (85.29)	51 (62.96)		
SSRS (points)			6.349	.012
<33 (n = 91)	49 (72.06)	42 (51.85)		
≥33 (n = 58)	19 (27.94)	39 (48.15)		
Family cohesion (points)			5.053	.025
<45 (n = 86)	46 (67.65)	40 (49.38)		
≥45 (n = 63)	22 (32.35)	41 (50.62)		
Family adaptability (points)			4.763	.029
<40 (n = 91)	48 (70.59)	43 (53.09)		
≥40 (n = 58)	20 (29.41)	38 (46.91)		

FoP = fear of disease progression, PSQI = Pittsburgh Sleep Quality Index, SSRS = Social Support Rating Scale.

### 3.7. Independent predictors of FoP: multivariate analysis

A multivariate analysis model identified significant independent predictors for FoP among stroke patients. Factors increasing the odds included greater neurological deficit (OR = 4.089, 95% confidence interval [CI]: 1.546–10.812), more comorbidities (OR = 6.223, 95% CI: 2.332–16.609), being unemployed (OR = 2.658, 95% CI: 1.151–6.138), and higher PSQI scores (OR = 4.430, 95% CI: 1.677–11.700). Factors decreasing the odds were higher age (OR = 0.373, 95% CI: 0.160–0.866) and greater SSRS scores (OR = 0.244, 95% CI: 0.098–0.606). Family cohesion and adaptability did not demonstrate a statistically significant association with the outcome (*P* > .05; Table 6).

**Table 6:** Independent predictors of fear of disease progression in stroke patients from multivariate analysis.

Indicators	*B*	SE	Wald	*P*	OR	95% CI
Age (yr)	−0.987	0.431	5.259	.002	0.373	0.160–0.866
Neurological deficit severity	1.408	0.496	8.059	.005	4.089	1.546–10.812
Comorbidities	1.828	0.501	13.323	<.001	6.223	2.332–16.609
Occupational status	0.978	0.427	5.244	.022	2.658	1.151–6.138
PSQI (points)	1.488	0.495	9.024	.003	4.430	1.677–11.700
SSRS (points)	−1.409	0.464	9.243	.002	0.244	0.098–0.606
Family cohesion (points)	−0.671	0.427	2.471	.116	0.511	0.222–1.180
Family adaptability (points)	−0.526	0.435	1.461	.227	0.591	0.252–1.387

CI = confidence interval, OR = odds ratio, PSQI = Pittsburgh Sleep Quality Index, SE = standard error, SSRS = Social Support Rating Scale.

## 4. Discussion

In this investigation involving 149 stroke patients, 45.64% exhibited a clinically significant FoP (FoP-Q-SF: 32.32 ± 7.90), a rate comparable to the 46.70% found by Guan et al^[17]^ According to Fan et al,^[18]^ such fear mediates the effect of adult attachment on quality of life, underscoring the clinical value of addressing FoP to improve outcomes, especially for those with high attachment avoidance. Sleep evaluation showed a mean PSQI score of 9.67 ± 2.00, with major impairments in sleep efficiency, daytime function, and sleep disturbances. Existing literature consistently associates sleep with neurological health. Sleep abnormalities like insomnia, fragmentation, circadian disruptions, and extreme sleep duration have been implicated in compromised brain health and may hinder poststroke rehabilitation.^[19]^ Furthermore, 1 observational and Mendelian randomization study identified insomnia and excessive daytime somnolence (frequent napping and dozing) as potential stroke risk factors.^[20]^ Thus, promoting healthy sleep may be beneficial both in stroke prevention and in fostering recovery. Yet, a direct investigation into the interplay between sleep quality and FoP in stroke survivors is currently lacking.

Further analysis revealed a predominantly moderate level of social support (97.99%). The mean SSRS score was 31.31 ± 4.11 points, aligning with reports by Sun et al.^[21]^ Our results are further complemented by Zhang et al,^[22]^ who demonstrated a strong link between social support and depression in ischemic stroke inpatients, mediated by fatigue levels. Additionally, measured family cohesion and adaptability averaged 43.22 ± 6.54 and 38.34 ± 6.43 points, respectively. Furthermore, significant correlations existed where FoP correlated positively with sleep disturbances, yet inversely with social support and family cohesion/adaptability. Physiologically, this fear may sustain elevated stress, potentially dysregulating the adrenal medullary hormone system, hypothalamic–pituitary–adrenal axis, and sympathetic nervous system. These systems’ hyperactivity can prevent patients from achieving deep sleep, thus reducing sleep quality.^[23]^ Conversely, supportive social and family relationships, providing both information and emotional comfort, can reduce patient stress and thus mitigate FoP. Corroborating this, a study on chronic heart failure patients^[24]^ has found a direct positive link between FoP and sleep disorders. Research in stroke populations has further identified sleep quality and social support as mediators in the relationship between perceived stress and depression,^[25]^ emphasizing the value of improving sleep and fostering supportive networks.

Finally, to identify independent predictors of FoP in stroke patients, we conducted univariate screening followed by multivariate regression modeling. Protective factors included age ≥ 60 years and strong social support, while moderate stroke severity, ≥2 comorbidities, unemployment, and sleep disturbances heightened FoP risk. With greater age (≥60) often comes greater psychological maturity and resilience, mitigating disease-related fears. Adequate social support provides practical resources and emotional sustenance that encourage a more composed and optimistic disease perspective. However, heavier illness burden and greater suffering, as seen in moderate stroke and multimorbidity, increase perceived threat. Unemployment limits socioeconomic resources, reducing one’s capacity to manage sudden health crises and associated burdens, thereby aggravating such fears. Chronic physical and mental exhaustion from sleep disorders erodes the cognitive and emotional resilience needed to cope with stressors, predisposing individuals to heightened disease-related fears. As documented by Lin et al,^[26]^ social support serves as an intermediary in the connection between symptom burden and FoP in stroke patients. Their study further highlighted unemployment, high symptom burden, and limited social support as contributors to heightened FoP, which aligns with our findings. Another research on cardiac patients has similarly identified younger age and comorbidities as risk factors for FoP, mirroring the associations observed here.

Based on the above results, a targeted intervention strategy can be formulated as follows: Initially, health education via specialized lectures or official account articles will help patients develop evidence-based disease understanding and improve perceived controllability. Subsequently, psychological assistance should address unresolved doubts and potential emotional conflicts; through attentive listening and structured counseling, patients may receive affirmative support to reduce psychological distress and pressure. Early rehabilitation guidance is recommended for moderate stroke patients to promote functional independence. In cases involving multiple comorbidities, a comanagement handbook can be provided to educate them on targeted strategies for each condition. To enhance self-efficacy and attenuate FoP, incentives can be introduced when patients show substantial improvement in managing their comorbidities. For jobless individuals, vocational potential will be assessed before discharge, with referrals to community-based job support services to aid occupational reintegration. To tackle sleep issues, implementing codeveloped activity-rest schedules will promote adherence to daily rehabilitation tasks and resynchronize the sleep-wake cycle. Additional measures, such as curtailing afternoon naps to a maximum of 2 hours and moderating evening stimulants/fluids.

Several limitations should be acknowledged. First, this was a single-center cross-sectional study conducted in a tertiary hospital in China, which may limit the generalizability of the findings to other regions or healthcare settings. Second, although the sample size met the minimum statistical requirements, it remains modest and may limit the detection of small effect sizes. Third, the cross-sectional design precludes causal inference between FoP and its associated factors. Therefore, conclusions should be interpreted cautiously, and longitudinal multicenter studies are warranted to validate these findings.

Furthermore, assessments were conducted 48 to 72 hours after admission, a period during which patients may still experience acute psychological stress related to hospitalization and recent neurological events. This timing could have influenced reported FoP and sleep quality levels, potentially introducing variability. Future studies may consider longitudinal assessments to evaluate dynamic changes over time.

## 5. Conclusion

Conclusively, clinically significant FoP is prevalent among stroke survivors, with notable correlations to sleep disturbances, social support levels, and family dynamics. Several factors increase the likelihood of significant FoP: age below 60, moderate stroke severity, ≥2 comorbidities, unemployment, insufficient social support, and sleep disorders.

## Author contributions

**Conceptualization:** Xu Liu, Lianhua Qin, Jieping Zhong.

**Data curation:** Xu Liu, Lianhua Qin, Jieping Zhong.

**Formal analysis:** Xu Liu, Lianhua Qin, Jieping Zhong.

**Funding acquisition:** Xu Liu, Lianhua Qin, Jieping Zhong.

**Investigation:** Xu Liu, Jieping Zhong.

**Writing – original draft:** Jieping Zhong.

**Writing – review & editing:** Jieping Zhong.
